# Machine Learning and Explainable Artificial Intelligence Using Counterfactual Explanations for Evaluating Posture Parameters

**DOI:** 10.3390/bioengineering10050511

**Published:** 2023-04-24

**Authors:** Carlo Dindorf, Oliver Ludwig, Steven Simon, Stephan Becker, Michael Fröhlich

**Affiliations:** Department of Sport Science, Rheinland-Pfälzische Technische Universität Kaiserslautern-Landau (RPTU), 67663 Kaiserslautern, Germany

**Keywords:** biomechanics, posture, hyperlordosis, hyperkyphosis, machine learning, artificial intelligence, explainable artificial intelligence, human-in-the-loop, confident learning, label errors

## Abstract

Postural deficits such as hyperlordosis (hollow back) or hyperkyphosis (hunchback) are relevant health issues. Diagnoses depend on the experience of the examiner and are, therefore, often subjective and prone to errors. Machine learning (ML) methods in combination with explainable artificial intelligence (XAI) tools have proven useful for providing an objective, data-based orientation. However, only a few works have considered posture parameters, leaving the potential for more human-friendly XAI interpretations still untouched. Therefore, the present work proposes an objective, data-driven ML system for medical decision support that enables especially human-friendly interpretations using counterfactual explanations (CFs). The posture data for 1151 subjects were recorded by means of stereophotogrammetry. An expert-based classification of the subjects regarding the presence of hyperlordosis or hyperkyphosis was initially performed. Using a Gaussian progress classifier, the models were trained and interpreted using CFs. The label errors were flagged and re-evaluated using confident learning. Very good classification performances for both hyperlordosis and hyperkyphosis were found, whereby the re-evaluation and correction of the test labels led to a significant improvement (M_PRAUC_ = 0.97). A statistical evaluation showed that the CFs seemed to be plausible, in general. In the context of personalized medicine, the present study’s approach could be of importance for reducing diagnostic errors and thereby improving the individual adaptation of therapeutic measures. Likewise, it could be a basis for the development of apps for preventive posture assessment.

## 1. Introduction

Promising potentials for objectified, data-based support through the integration of artificial intelligence, and its subcategories of machine learning (ML) and deep learning for data interpretation, have been shown for the healthcare sector in numerous studies. It has been demonstrated that these techniques are beneficial for analyzing complex and multivariate data; finding discriminative, class-specific differences; and ultimately providing objective, data-based decision support to medical practitioners [1,2]. Furthermore, an advantage over the commonly used inference-based statistical analysis methods has been reported [3,4]. It has been shown that ML-based systems even surpass human guidance in disease detection [5,6]. In addition, a reduction in false-positive mistakes and the mitigation of different experience levels of medical practitioners have been reported [7]. In the context of concrete biomechanical use cases, ML has proven useful in the diagnosis of gait disorders [8,9], the recognition of human activities [10], age-related assessments [11,12], and the optimization of the rehabilitation phase [13]. Various biomedical diseases have been considered, e.g., after a stroke [8], in Parkinson’s disease [9], in osteoarthritis [14], and in total hip arthroplasty [15]. However, regarding the application of ML methods for the evaluation of posture parameters, little research has been conducted [16].

A common way to check a person’s posture is to assess the back contour through a visual inspection. However, this procedure is susceptible to subjectivity and potential errors. A comparative study where 28 chiropractors, physical therapists, rheumatologists, and orthopedic surgeons evaluated the posture of subjects from lateral photographs found that the intra-rater reliability was only moderate (kappa = 0.50), and the inter-rater reliability was weak (kappa = 0.16) [17]. There is, therefore, a great need for research to support medical diagnostics with data-based, yet transparent, ML methods. This has recently led to the development of smartphone apps that assess posture in a semi-automated way [18]. In the context of medical image processing, e.g., in the field of orthopedics [19] or tumor detection [20], ML methods have shown very good results regarding the detection of abnormalities. Although very promising results in medical image data are present, compared to the medical image domain, relatively few works can be found in the context of biomechanical data using ML methods [21], mainly focusing on dynamic gait data (e.g., [2,22]). Some studies have already shown promising results using machine learning for generating objective, data-based orientations for static posture evaluation [23].

On the other hand, the relevance of posture measurements in the field of prevention and rehabilitation is well known [24,25]. Especially in the transition area from (correctable) postural weaknesses to pathological postural damage, there is often no consensus between individual examiners [17]. Reference values are known [26,27,28], but the transition to pathological postural variations requiring therapy is fluid. Therefore, possible support for medical diagnostics via computer-assisted AI methods is valuable. Methods that support these diagnostics while allowing assessments that are comprehensible to the user may therefore be of great medical benefit. Overall, the transfer and further development of existing methods in the context of biomechanical data, such as postural data here, is therefore still an important, open research field.

Due to the difficulty and error-proneness of objectively assessing posture by experts, it can be concluded that training, as well as test posture data for ML, might be negatively affected in terms of wrongly assigned labels by experts. On the one hand, this negatively affects the training process of an ML classifier, and, on the other hand, the true performance of the test data is possibly underestimated. This problem is known even for test data from benchmark datasets (e.g., MNIST, ImageNet) [29]. A re-assessment of class labels is often not possible, simply because the datasets are large, and, therefore, not all cases can be re-examined economically. A recently described approach for dealing with these problems is *confident learning* for estimating uncertainty in dataset labels [30]. The approach enables both the supervised training of a model for training data with incorrect labels and the identification of possible errors in the test data, which can then be re-evaluated by experts and thus corrected. Although there are promising results from confident learning [29,30,31,32], and the characteristics of biomechanical expert evaluations, which can be accompanied by errors, highlight the importance of such approaches, no work is known to the best of the authors’ knowledge that has applied confident learning in the context of biomechanical or sports science issues.

Regarding the use of ML models, the model’s opacity often makes it difficult for users to trust and understand its decisions [28]. Such a lack of transparency violates the requirements of the European General Data Protection Regulation (GDPR, EU 2016/679) [33], which greatly limits the practicality of using the model in clinical settings [34]. Most biomechanical studies have not overcome this limitation and have used ML as black-box models [8,9,14]. Recent advances in *explainable artificial intelligence* (XAI) have made it possible to make ML more and more applicable in practical clinical contexts, for example, in the biomechanical domain [22,35]. XAI provides various methods for increasing the trustworthiness and transparency of black box models [36], such as local interpretable model agnostic explanations (LIME) [37], Shapley additive explanations (SHAP) [38], and deep learning important features (DeepLIFT) [39]. The use of XAI has proven especially valuable in understanding personalized differences in pathology, such as in monitoring pre- and post-operative therapy measures, and is thus highly relevant to the field of personalized medicine [22].

Although these works have shown interesting perspectives, local interpretations have so far mainly focused on a few XAI methods in the biomedical domain, e.g., LIME [35], or layer-wise relevance propagation [2]. Furthermore, until now, these methods have not shown to what extent changes in the implemented features would have an influence on the model prediction. This, however, would be highly relevant, both in the context of good comprehensibility and in terms of the planning of therapy measures that normally depend on the classification of a human examiner.

*Counterfactual explanations* (CFs), an XAI tool, could be a way to address these aspects, which, to the best of the authors’ knowledge, has not yet found its way into the biomedical context. CFs examine which features would need to be changed to achieve a desired prediction. Since human posture is multifactorial, i.e., a large number of individual posture parameters (e.g., depth of lumbar lordosis, forward tilt of the pelvis, degree of thoracic kyphosis) are included in the summary assessment by a physician, it would be interesting to know for which combinations and expressions of these individual parameters he would assess the posture as correct. In the context of this binary classification problem of a posture assessment (“good” or “weak”, which means “no therapy” or “therapy”), this could mean that, for a subject classified with an 80% probability as pathologic: “if we could improve the pelvic tilt by X degrees, the patient would be classified as not having poor posture with a probability of 80%”, whereby individual personal characteristics (e.g., gender, age) could be additionally included. By providing explanations in this way (explanations contrastive to the instance of interest) and usually focusing on a small number of features to change, CFs are particularly human-friendly explanations [40].

Due to the above-mentioned research deficits, the aim of the present work was twofold for using the posture data of subjects with hyperkyphosis or hyperlordosis, as well as healthy subjects: First, we wanted to evaluate the general modeling abilities and check if it is possible to classify the presence of hyperkyphosis or hyperlordosis to give an objective, data-based orientation. In parallel, we wanted to evaluate confident learning for model training, as well as to test data label error identification, and check if the reevaluation of flagged test labels and a potential correction improves the performance of the model. Second, we wanted to analyze if CFs add useful insights into the trained models and provide plausible suggestions for the improvement of the parameters in biomechanical terms.

## 2. Materials and Methods

### 2.1. Subjects and Data Acquisition

The data were collected from 1151 subjects. The exclusion criteria were chronic diseases of the spine or the musculoskeletal system, a previous spinal surgery, leg length discrepancies greater than 5 mm, and dizziness. Two subjects with missing data were excluded from further analyses, resulting in a total of 1149 subjects who were used for the further calculations (sex: 691 male, 458 female; age: 35.13 ± 15.91 years; weight: 73.86 ± 17.97 kg; height: 172.97 ± 10.17 cm). No outliers were removed. The study was approved by the Ethical Committee of the university (Saarland University: UdS 15-6-08; RPTU: 23-57) and met the criteria of the Declaration of Helsinki [41]. All the participants signed informed consent forms, including permission to publish the results of the study. In the case of minors, the consent of the legal guardian was obtained.

The examinations were conducted with a mobile scanner (Bodybalance 4D, Paromed Bodybalance GmbH, Neubeuern, Germany). The test subjects stood in a habitual position with a bare upper body (women in bras) at a distance of 2.30 m from the device. The examiner had previously marked the following anatomical landmarks with white marker dots (diameter of 12 mm): the spinous process of the 7th cervical vertebra (C7); the vertices of the cervical, thoracic, and lumbar spine curvatures; the spinous process of the 1st sacral vertebra (S1); the posterior superior iliac spine (PSIS); and the tips of the shoulder blades. Each scan was performed four times, and the obtained values were averaged. The anatomical landmarks were automatically recognized by the system and manually checked and confirmed again by the examiner. 

**Figure 1:**
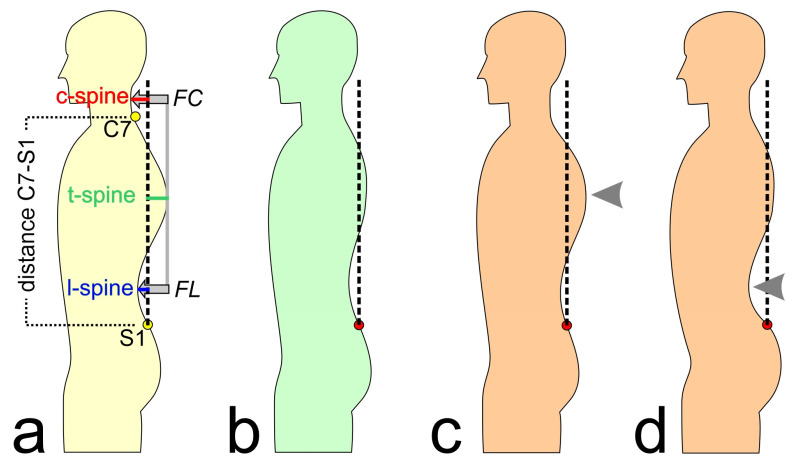
(**a**) Explanation of the analyzed posture parameters; C7: 7th cervical vertebra, S1: 1st lumbar vertebra, FC: flèche cervicale, and FL: flèche lombaire. (**b**) Normal posture; the dashed line represents the perpendicular axis through S1. (**c**) Increased thoracic kyphosis (hyperkyphosis, or “hunchback”). (**d**) Increased lumbar lordosis (hyperlordosis, or “hollow back”). Arrows mark the posture deviations (**c**,**d**).

The available features are presented and described in Table 1 and Figure 1. The subjects’ characteristics were included as features for the modeling. Based on the measured raw data for *distance C7-S1*, *c-spine*, *t-spine*, and *l-spine* (see Figure 1), the feature kyphosis index (KI), flèche cervicale (FC), and flèche lombaire (FL) were calculated, as they are commonly used for posture evaluations [42,43]. Further, these calculated features were normalized by the distance between C7 and S1 (corresponding to the subjects’ trunk heights) to allow better comparability between the subjects (hereinafter abbreviated as KI%, FC%, and FL%).

Based on the measurements and on a visual inspection of the subjects, four experienced biomedical experts performed a classification of the subjects regarding the presence of hyperkyphosis of the thoracic spine or hyperlordosis of the lumbar spine, with each subject being evaluated by one of the four raters only. All the investigators had many years of experience in the field of posture analysis and worked according to the same assessment standards. Accordingly, 420 subjects (36.56%) showed hyperkyphosis, and 411 (35.77%) showed hyperlordosis.

### 2.2. Feature Set and Modelling

When interpreting black box models, the influence of different data representations on both the classification accuracy and interpretability must be kept in mind. It is evident that ML models can only be interpreted as well as their features. Even simple, highly interpretable model types can be difficult or impossible to understand if no human-interpretable features are used [44]. In addition, different levels of background knowledge and expertise must be taken into account when developing interpretable features in order to optimally lock onto the existing knowledge of the users; otherwise, the features quickly become difficult to understand again for specific target groups [1]. Consequently, for predicting the presence of hyperkyphosis and hyperlordosis, interpretable features that are of high relevance in practice, as well as supported by former studies, were selected for the modeling. Therefore, regarding the reported age- and gender-related effects on posture parameters, as well as the high practical relevance and comparability of the height-normalized indices [26], the features of gender, age, KI%, FC%, and FL% were used for the modeling. For an evaluation of the selected feature set, the modeling results were compared with those of models trained on all 15 features presented in Table 1. Inconsistent results are reported regarding BMI as an influencing factor for posture [26,27,28]. However, in order not to ignore a possible influence a priori, BMI was included as a feature for the evaluation of the selected feature set.

For the classification of hyperlordosis and hyperkyphosis, a one vs. rest multi-label strategy was followed. Thus, one classifier was fitted per class against all the other classes. The model training was integrated into a stratified five-fold cross-validation procedure (with the folds preserving the percentage of the samples for each class) to obtain an unbiased accuracy score. For each fold, the data were split by approximately 80% into training and 20% into test data. The test data were completely separated and only used for testing. Due to an imbalanced class distribution, the *synthetic minority over-sampling technique for nominal and continuous features* (SMOTENC) was applied to create training data with balanced classes using the Python library “imbalanced-learn” [45].

A *Gaussian process classifier* was used for the classification, as research has shown its ability to predict well-calibrated probabilities and its superior performance compared to logistic regression [46]. Further, the Gaussian process classifier has been successfully used in medical studies [47,48]. For the model implementation, the *scikit-learn* Python library [49] was used with the hyperparameters set to the default values. The data scaling was performed by removing the mean and scaling to the unit variance, based on the respective training dataset for each fold. For an evaluation of the model selection, logistic regression was applied, as it is known to be an interpretable model.

Uncertainties were reported as classification probabilities. Due to imbalanced data, *precision–recall curves* and the *precision–recall area under the curve* (PRAUC) metric were reported. The probabilities were transformed into crisp values by using the 0.5 threshold. The respective accuracies were reported with the confusion matrix, as well as the F1 score and the Matthews correlation coefficient (MCC) due to the imbalanced classes. The calculations were performed in Python (Python Software Foundation, Wilmington, DE, USA).

### 2.3. Confident Learning, Interpretation, and Evaluation

Potentially wrong test labels were flagged using the Python confident learning library *cleanlab* [32]. Using cleanlab, and on the basis of the Gaussian process classifier models directly trained on each training fold dataset during the cross-validation, the potentially wrong test labels were automatically flagged. The flagged test data were then re-labeled by experienced experts using a digital survey. Additionally, the age and gender of the subject to be re-evaluated were presented to the experts alongside the data. The original class labels were hidden. In the first step, two experts were asked for their assessment of all the flagged subjects. In the event of an inconsistent assessment, a third expert was also called in, and the majority vote was selected as the final class label.

As label errors also seemed likely in the training data of each fold, confident learning during the training procedure was additionally applied. To evaluate the influence of the possible correction of the test labels, as well as the confident learning during the training process, the classification results were therefore presented for the following scenarios:

1. Test performance on the given test labels using the Gaussian process classifier;
2. Test performance on the corrected test labels using the Gaussian process classifier;
3. Test performance on the given test labels using the Gaussian process classifier + confident learning on the training data;
4. Test performance on the corrected test labels using the Gaussian process classifier + confident learning on the training data.

Local interpretations (interpretations of individual instances/subjects) of the trained models were performed using CFs with the Python library *diverse counterfactual explanations* (DiCE). Studies have shown promising results for using this library to generate CFs [50,51]. The parameters, including *proximity* and *diversity weights*, were set to the default values. To capture the variability (also called diversity) of the CFs, ten explanations were generated for each instance that needed to be explained. Therefore, the data from each test set with the respective calibrated models were used. As the subject characteristics (age, gender) were impossible to change in a real setting, feature changes were allowed only for the posture parameters, which might be possible to change through therapy measures.

Additionally to the local interpretations, global interpretations (interpretations over multiple instances/subjects) were reported through the aggregation of the local interpretations, similar to [35]. Thus, the ten CFs per subject were aggregated for each feature using the median. For the global interpretations, the data for wrongly predicted instances according to the crisp values were excluded.

For an evaluation of the CFs in terms of plausibility in biomechanical terms, the global changes between the subjects with postural deficits and global CFs were statistically checked. Further, the global changes were also checked if the CFs for the subjects with hyperkyphosis and hyperlordosis met the characteristics of the healthy subjects. Therefore, the aggregated data used for the global interpretations were used, and a Mann–Whitney U test was applied as a non-parametric test to check for potential differences. The statistical tests were performed with the Python library SciPy [52]. The *p*-values were compared to an alpha level of 0.05. All the calculations were performed on a Katana GF66 11UG-220 computer (MSI, Micro-Star International Co. Ltd., Taiwan, China).

## 3. Results

### 3.1. Re-Evaluation Results

Originally, of the 1149 subjects, 420 showed hyperkyphosis, and 411 showed hyperlordosis. After a re-evaluation and the correction of the flagged instances, 424 showed hyperkyphosis, and 423 showed hyperlordosis. The results of the re-evaluation are presented in Table 2. For the classification of hyperkyphosis, more flagged labels, a larger disagreement among the raters, and more actually corrected labels were found compared to the classification of hyperlordosis.

**Figure 2:**
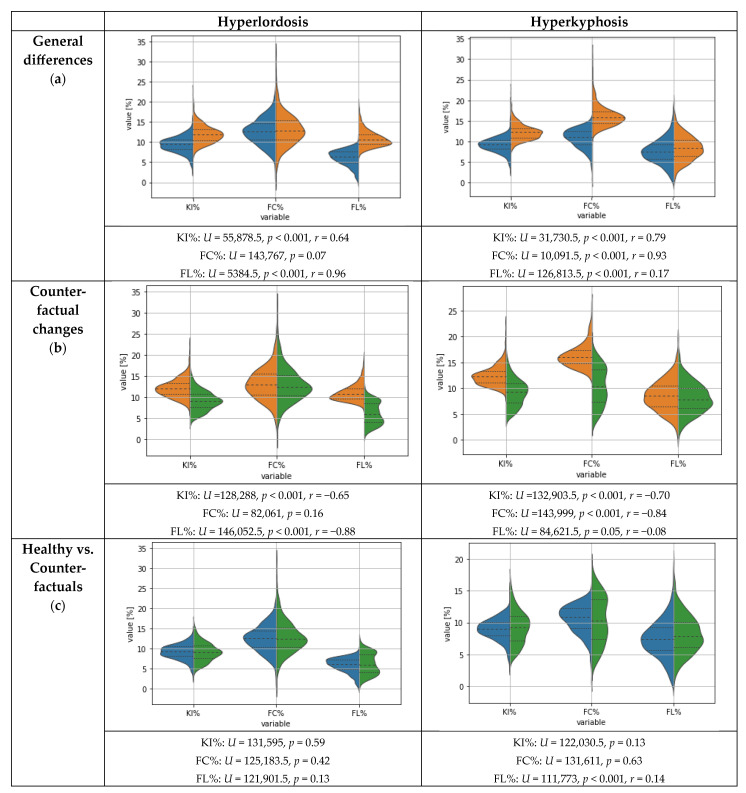
Violin plots for the posture parameters. Dashed lines represent median and quartiles. Age and gender are not displayed as features since they are impossible to change. Blue = healthy subjects without postural deficits; orange = subjects with hyperkyphosis or hyperlordosis; green = global counterfactual explanations, suggesting necessary feature changes for the subjects with postural deficits to be classified as healthy subjects by means of the ML algorithm.

Figure 2 (upper plots) shows the general differences, including the statistical test results, between the features for the subjects with and without hyperlordosis or hyperkyphosis and the healthy subjects after correcting the flagged test labels. The statistical differences between the healthy subjects and the subjects with hyperlordosis were mainly observable for the features KI% and FL%. The subjects with hyperkyphosis differed from the healthy subjects for the features KI% and FC%. 

### 3.2. Modeling Results

**Figure 3:**
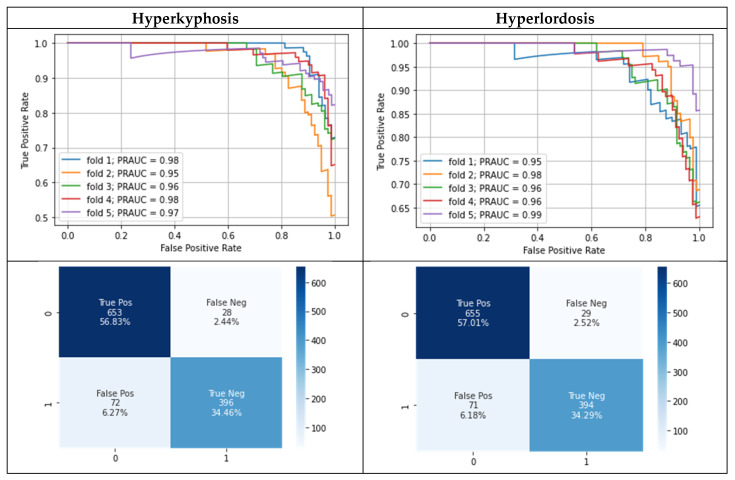
Precision–recall curves during five-fold cross-validation (each curve represents results for test data of one cross-validation fold) as well as a separate confusion matrix for hyperkyphosis and hyperlordosis using confident learning and the corrected test labels. Lower row: 0 = healthy, 1 = postural deficit.

The model training took about 2 min per cross-validation fold without confident learning and about 3 min with confident learning. Table 3 and Figure 3 show the modeling results separately for predicting the presence of hyperlordosis and hyperkyphosis. Approximately the same modeling performance for hyperlordosis and hyperkyphosis was present. The best modeling results were achieved after correcting the flagged test labels, whereas an improvement was observable compared to the use of the given test labels. However, no difference in the mean area under the precision–recall curve (M_PRAUC_) after correcting the flagged test labels was present when using the potentially wrongly labeled training data for the model training with confident learning.

Using the selected feature set consisting of the features gender, age, KI%, FC%, and FL% showed superior performance compared to using all available features with confident learning and the corrected test labels for both hyperkyphosis (M_PRAUC_ = 0.90 ± 0.04, M_F1_ = 0.86 ± 0.02, M_MCC_ = 0.77 ± 0.04) and hyperlordosis (M_PRAUC_ = 0.91 ± 0.02, M_F1_ = 0.88 ± 0.02, M_MCC_ = 0.80 ± 0.03). Logistic regression using the selected features resulted in approximately the same performance for the classification of hyperlordosis (M_PRAUC_ = 0.97 ± 0.01, M_F1_ = 0.89 ± 0.03, M_MCC_ = 0.83 ± 0.05), but a slightly reduced performance compared with the Gaussian process classifier for the classification of the presence of hyperkyphosis (M_PRAUC_ = 0.95 ± 0.01, M_F1_ = 0.87 ± 0.02, M_MCC_ = 0.80 ± 0.03).

### 3.3. Results for Counterfactual Explanations

**Figure 4:**
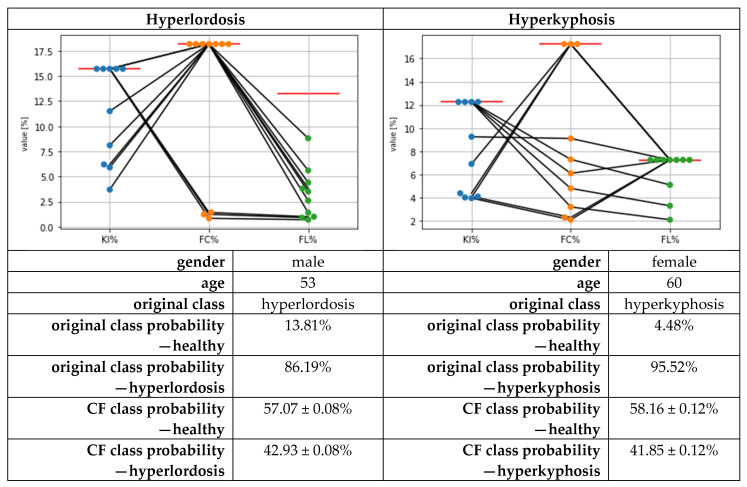
Exemplary local counterfactual explanations (CFs) for a subject with hyperlordosis (**left**) and a subject with hyperkyphosis (**right**) when changing the class membership to the class of healthy subjects. Each dot per feature represents one out of ten suggested feature values. Red horizontal lines represent the original feature values mapping the postural deficits.

The calculation of the CFs using the DiCE library took about 10 min for each cross-validation fold’s test data. The exemplary local results for two subjects regarding the CFs are presented in Figure 4. Based on these results for hyperlordosis, the CFs mainly suggested reducing KI% and FL%, compared to the given feature values, and keeping the FC% feature value. In three of the ten cases, the CFs suggested keeping the KI% value and changing the FC% and FL% values. For hyperkyphosis, the changes were mainly suggested compared to the given feature values for KI% and FC%, and only in three cases for FL%.

The exemplary CF results are in line with the global feature changes for inverting the class membership of the subjects with hyperkyphosis and hyperlordosis (see Figure 1, middle plots). On a statistical basis, for hyperlordosis, the greatest changes were observed for FL%, followed by KI%. For hyperkyphosis, statistically significant changes were present in descending effect size for FC%, KI%, and FL%. However, the changes for FL% were small, with *p* = 0.05 at the alpha-level threshold.

The global results for the CFs inverting the class labels in the presence of hyperlordosis and hyperkyphosis are presented and compared with the original feature values of the healthy subjects in Figure 2 (lower plots). Visually, for both hyperlordosis and hyperkyphosis, differing distributions could be observed; however, only small differences were observed in the median values. A statistical comparison by means of a Mann–Whitney U test showed that the CFs did not differ from the healthy group characteristics for all regarded features of the hyperlordosis class. However, for the CFs of the hyperkyphosis class, the feature FL% differed from the healthy group characteristics, but with a small effect size, according to Cohen [53]. No further differences were found for hyperkyphosis.

## 4. Discussion

The present results show that it is possible to classify the presence of hyperlordosis or hyperkyphosis based on postural data measured using stereophotogrammetry by means of ML. The use of confident learning to show possible class label errors in the test set, and the re-evaluation and correction of the respective cases by experts, showed that the original labels of the test data were partially incorrect. After correcting the class labels for both hyperlordosis and hyperkyphosis, the best mean PRAUC value of 0.97 was achieved. The erroneous test labels, therefore, led to the actual performance of the model being underestimated.

In the present case of the ML-based classification of hyperlordosis and hyperkyphosis, around 10% of the test labels were incorrect. In particular, when the datasets were not labeled by combining the expert judgments of several people, as was also the case in the present dataset, the described approach could help to identify errors in the existing data without having to check all the data samples again, which is, in many cases, not feasible for economic reasons. Although the results highlight the benefits of using confident learning to identify potentially mislabeled test-set labels, no performance benefits were found when using confident learning for model training with partially mislabeled training data.

Since feature extraction is an important step to improve the accuracy of a model, avoid overfitting, reduce the computing power, and improve the interpretability [54], a reduction in the number of suitable features should be aimed for. With regards to interpretability, especially in relation to previous research and existing knowledge, expert-based features, which are common in practice and reported in the literature, proved to be superior [35,44]. The results with selected, interpretable, and practice-relevant features led to improved classification results in the study compared to the use of all the available features. Nevertheless, in this context, a possible a priori loss of information due to feature selection should be critically discussed, which is particularly related to non-data-based selections [1]. However, the potential a priori loss of information through expert-based feature construction and selection appears to be low overall, since the selected features achieved improved classification results compared to the use of available features as the model input. Therefore, it can be assumed that the present expert-based feature set is highly suitable and superior to the use of the whole set of available features.

According to [32], the criteria for good CFs include the following: (a) a CF with the predefined class prediction can be generated; (b) a CF should be close to the instance in terms of the feature values, and it should change as few features as possible; (c) several different CFs should be provided; and (d) a CF should have probable or realistic characteristic values. For evaluation, these aspects are discussed below:

(a) In this study, ten different CFs could be found for each person. Consequently, the results show that it was, in general, possible to find CFs for the specified task. (b) Considering the global feature changes, the CFs were relatively close to the original feature values, and a maximum of two features was dominantly varied per class. The changes appeared to be necessary to change the class membership, since the healthy subjects and the subjects with hyperkyphosis and hyperlordosis, according to the results of this study and other research [26], showed differences in their respective features. Accordingly, the analysis of the exemplary local CFs also showed that these were relatively close to the original characteristic values, and that individual characteristic changes predominated. Overall, this corresponds to the criterion mentioned.

In the present study, the proximity and diversity were set to the default values of scikit-learn. Depending on the area of application, further tuning of the parameters can be useful. For example, increasing the proximity weight might result in features that are closer to the original query instance and less diverse.

(c) Ten different CFs were given for each instance, which again speaks to the fulfillment of the criterion. However, providing multiple solutions is both advantageous and disadvantageous. The question remains of how to find a reasonable, context-relevant, and meaningful explanation from all the explanations provided. A possible approach could be either the definition of context-specific external criteria to select the most appropriate CF or an expert-based selection based on prior knowledge and suitability for individual subject characteristics.

(d) Looking at the features that were globally modified to change the class prediction of the subjects with postural deficits, it can be seen that differences between the healthy subjects and the subjects with hyperlordosis were mainly observable for the features KI% and FL%. The subjects with hyperkyphosis differed from the healthy subjects by the features KI% and FC%. This is consistent with the differences reported in the literature for hyperkyphosis and hyperlordosis [55], as well as the statistical comparison of healthy subjects and the subjects with postural deficits in this study. The XAI interpretations thus appear plausible overall.

The results show that the CFs, which changed the characteristics of the subjects with postural deficits towards the healthy subjects with regard to the feature, FL%, for hyperkyphosis, did not agree with the feature values of the healthy group according to the Mann–Whitney U test. However, the small effect size did not appear to indicate a greater implausibility. No statistical differences were found for any of the other features, which in turn speaks to the general plausibility of the CFs.

On closer inspection, the distributions of the trait values did not match exactly, but the values of the CFs appeared to be closely related to the feature values in the distribution of the healthy subjects and were, therefore, at least realistic. Thus, it seems likely that CFs can meaningfully shift the class affiliation of individuals with postural deficits based on the postural parameters used for healthy individuals and small possible feature changes. Since this is one of the first works in this field without sufficient comparative studies being available, it is necessary to further evaluate these findings with future studies. Furthermore, the optimization of the parameters proximity and diversity could also have the potential to better correspond with the actual characteristics of healthy people.

Based on [20], the black box problem (a) and the problem of labeling the data (b) can be characterized as central challenges when using AI with biomechanical data. In the present work, contributions were made to solving the problem in (a), which, in contrast to other methods from the XAI area, is particularly user-friendly, and the problem in (b) through label error detection. In the present study, CFs were used as an XAI tool for interpretation. However, it should be noted that it has not been analyzed intensively whether other XAI methods match with the results found for the CFs and, thus, support the local suggestions. In general, the agreement between different XAI methods and the XAI results of different classifiers is little addressed, whereas more or less strong variations of the XAI results are to be expected [22]. Therefore, future work should try to combine different XAI interpretation methods to generate more robust interpretations as an ensemble approach.

Although very good modeling results were obtained, there are several points to discuss that are related to the persistent modeling error and could help to further reduce it, e.g., the experimental design could be optimized to improve the class separation (development of an optimal experimental design). It should also be noted that logistic regression shows a reduced performance only in the classification of hyperkyphosis and, otherwise, has a similar model performance to the Gaussian process classifier. Since logistic regression is itself a very interpretable approach, it may also be useful, depending on the area of application, to use logistic regression only for classification and to interpret the model directly, rather than generating CFs. Nevertheless, there are also promising results that have been reported for the use of logistic regression in combination with CFs [56].

For the evaluation, the current study compared the statistical characteristics of the characteristic feature values of the healthy test subjects with the CFs, which suggests what the characteristics of the test subjects with hyperlordosis and hyperkyphosis should look like, so that they can be classified as healthy. For the global analysis, the ten CFs of each subject were aggregated to form a median, which might possibly eliminate the original relationships between the features. Consequently, for future works, another analysis that takes into account the relationships between the features could be the individual assessment of the local CFs by experts.

Summing it up, the presented approach combining confident learning with XAI might act as a data-driven, objective orientation for reducing expert-based errors of posture classifications regarding healthy characteristics or the presence of hyperkyphosis and hyperlordosis. As experts tend to show diverging results regarding the rating of human postures [17], the study’s approach might also hold the potential for reducing inter-rater differences. In addition, the class probabilities provided by the algorithm are superior to absolute class assignments for monitoring changes, and that may be useful for monitoring therapy progress, e.g., by examining the shift in classification probabilities towards the class of healthy subjects. In the context of personalized medicine, the local interpretations of the proposed approach could be of great use for the individual adaptation of therapeutic measures, since they include further influencing factors (here, age and gender), as well as individual initial conditions. The major advantages over existing works are that the proposed approach can flag errors in existing datasets and provides particularly human-friendly explanations of the classifications using CFs.

The present study has some limitations, which must be considered when interpreting the results and extending them to practical applications. First, it should be emphasized that the classification carried out by the algorithm for the objective detection of hyperkyphosis and hyperlordosis represents only a part of the overall assessment process. Posture is a multidimensional phenomenon. For its summary assessment, there are many factors (perpendicular distances, angles such as pelvic inclination, etc.) that can be considered for analysis, depending on the instrumental possibilities of the examiner and his experience. In the present work, we have limited ourselves to a few measured variables. On the one hand, this is of course a limitation; on the other hand, the values used have the great advantage that they can be measured easily and quickly. To measure the distance parameters FC and FL, it is not necessary to use expensive 3D measuring systems. These parameters can also be determined using simpler methods, for example, time-of-flight cameras, posture photographs, or even plumb bobs and rulers. Therefore, they are of particular relevance, which in turn we consider to be the strength of precisely these measurement values [26].

A further limitation is that the usability of Gaussian process models to large-scale datasets is limited, as it scales cubically and quadratically with the training data size [57]. Therefore, with an increasing dataset size, either other classifiers or adapted variations of the GP classifier (e.g., see [58]) should be considered for obtaining reasonable training times in practical applications. Another practical limitation is that the resulting models can only recognize characteristics for which they have been trained (here, hyperlordosis and hyperkyphosis) and are therefore pathology-dependent. Recently, interpretable, pathology-independent classifiers have been proposed to deal with this limitation [16,59].

Transferring the methodology of the present study to these classifiers could potentially create a powerful tool and could further increase the practical relevance of the ML methodology in biomechanical research. Future studies should include further anthropometric and postural parameters and help identify those most important for a clear diagnosis.

## 5. Conclusions

As experts tend to show diverging results regarding the rating of human posture, the proposed approach of combining confident learning with XAI using CFs proved useful as a data-driven, objective orientation for reducing inter-rater differences. The method used in this study could help in the development of apps that assess posture in an automated way.

## Figures and Tables

**Table 1:** Measured and calculated features; also see Figure 1.

Type	Feature	Description
subject characteristics	age	in years
	gender	male/female
	height	body height in cm
	weight	body weight in kg
	BMI	weight/height^2^
directly measured by system	distance C7–S1	vertical distance between the 7th cervical and the 1st sacral vertebrae in mm
	c-spine	horizontal distance between the apex of the cervical lordosis and the perpendicular axis through the 1st sacral vertebra in mm
	t-spine	horizontal distance between the apex of the thoracal kyphosis and the perpendicular axis through the 1st sacral vertebra in mm
	l-spine	horizontal distance between the apex of the lumbar lordosis and the perpendicular axis through the 1st sacral vertebra in mm
calculated features	KI	(FC-FL)/2
	FC	Absolute value of difference between c-spine and t-spine
	FL	Absolute value of difference between l-spine and t-spine
normalized features	KI%	KI × 100/distance C7–S1
	FC%	FC × 100/distance C7–S1
	FL%	FL × 100/distance C7–S1

**Table 2:** The results of the re-evaluation of the flagged labels, separated for the classification of hyperkyphosis and hyperlordosis, for the 1149 subjects of all cross-validation folds.

	Hyperkyphosis		Hyperlordosis	
	n	%	n	%
Highlighted labels	130	11.31%	110	9.57%
Agreement of the first two reviewers	94	72.31%	89	80.91%
Labels additionally assessed by a third expert	36	27.69%	21	19.09%
Highlighted labels corrected	112	86.15%	98	89.09%

**Table 3:** Classification results using the original data as well as confident learning and corrected test labels. Note: corrected labels were not used for model training. M_PRAUC_ = mean area under the precision–recall curve; M_F1_ = mean F1 score; M_MCC_ = mean Matthews correlation coefficient.

		Hyperkyphosis	Hyperlordosis
Test performance (on given test labels) using Gaussian process classifier	M_PRAUC_	0.80 ± 0.06	0.84 ± 0.05
	M_F1_	0.78 ± 0.03	0.77 ± 0.03
	M_MCC_	0.64 ± 0.05	0.63 ± 0.05
Test performance (on corrected test labels) using Gaussian process classifier	M_PRAUC_	0.97 ± 0.01	0.97 ± 0.01
	M_F1_	0.90 ± 0.03	0.88 ± 0.04
	M_MCC_	0.85 ± 0.05	0.82 ± 0.06
Test performance (on given test labels) using Gaussian process classifier (+confident learning on training data)	M_PRAUC_	0.78 ± 0.06	0.83 ± 0.04
	M_F1_	0.76 ± 0.04	0.77 ± 0.03
	M_MCC_	0.61 ± 0.05	0.64 ± 0.05
Test performance (on corrected test labels) using Gaussian process classifier (+confident learning on training data)	M_PRAUC_	0.97 ± 0.01	0.97 ± 0.02
	M_F1_	0.89 ± 0.04	0.89 ± 0.03
	M_MCC_	0.82 ± 0.06	0.82 ± 0.06

## Data Availability

The data are available if there is justified research interest.
